# Complete Labial Fusion Causing Urinary Retention and Pyo-Hematocolpos in a Female With Turner Syndrome

**DOI:** 10.7759/cureus.94986

**Published:** 2025-10-20

**Authors:** Georgios Androutsopoulos, Nikolaos Antonakopoulos, Georgios Michail, Georgios Katsougiannopoulos, Georgios Adonakis

**Affiliations:** 1 Department of Obstetrics and Gynecology, School of Health Sciences, University of Patras, Patras, GRC

**Keywords:** gonadal dysgenesis, hypoestrogenism, labial fusion, pyo-hematocolpos, turner syndrome, urinary retention

## Abstract

Turner syndrome, the most common chromosomal disorder in females, is associated with hypoestrogenism, which predisposes patients to genital tract atrophy and complications such as labial fusion. Although often asymptomatic, complete fusion may cause urinary obstruction, infection, and, in rare cases, life-threatening sepsis. We report a case of a 58-year-old female with Turner syndrome who presented with urinary retention, fever, and systemic signs of sepsis. Examination revealed complete labial fusion, obscuring the vaginal introitus, with imaging confirming hydro-pyo-hematocolpos. Laboratory findings were consistent with systemic inflammation and impaired renal function. Emergency surgical separation of the fused labia led to the drainage of purulent and hemorrhagic fluid, restoration of normal genital anatomy, and insertion of a Foley catheter. The patient was managed with broad-spectrum antibiotics, inotropes, intravenous fluids, and local estrogen therapy, achieving full recovery and discharge after 14 days.

Labial fusion in Turner syndrome results from chronic hypoestrogenism, recurrent inflammation, and mechanical irritation. While typically underdiagnosed, advanced cases can cause urinary retention, ascending infections, and pyocolpos. Surgical separation is an effective treatment modality, particularly in severe cases complicated by obstruction or infection, with postoperative estrogen therapy minimizing recurrence. This report highlights the need for heightened clinical vigilance for labial fusion in Turner syndrome patients. Early recognition and timely surgical intervention are essential to prevent severe complications, including hemato-pyocolpos and sepsis, and to improve quality of life.

## Introduction

Turner syndrome is the most prevalent sex chromosome disorder in females, caused by the complete or partial absence of the second X chromosome [1]. It occurs in approximately one in 2,500 live-born female infants. The most common phenotype is characterized by gonadal dysgenesis and short stature; however, the condition can involve multiple organ systems, including the cardiovascular, renal, endocrine, gastrointestinal, neurological, psychiatric, musculoskeletal, and dermatologic systems. Despite these widespread effects, most individuals with Turner syndrome have normal intelligence. [2].

A small proportion of females with Turner syndrome maintain normal ovarian function into early adulthood, and the majority have normal internal and external vaginal anatomy. Labial fusion is a common complication in Turner syndrome, attributed to the condition's hypoestrogenic state and characterized by thin, membranous adhesion of the labia [3,4]. Complete labial fusion may totally conceal the vaginal introitus, causing urinary retention [4]. Despite its potential to cause significant morbidity, labial fusion in individuals with Turner syndrome is often underdiagnosed. This is due to its typically asymptomatic early stages, frequent misattribution to other urogenital conditions, or a delay in patients seeking appropriate medical evaluation. We present a case of complete labial fusion causing urinary retention and pyo-hematocolpos in a female with Turner syndrome. By examining the underlying pathophysiological mechanisms and clinical implications, this report aims to underscore the necessity of heightened clinical vigilance, prompt recognition, and appropriate management of this rare condition.

## Case presentation

The patient was a 58-year-old female admitted to the emergency department of our hospital due to urinary retention and a fever up to 39 °C. Her recent medical history included abnormal urination stream, incomplete voiding and dribbling of urine, dysuria, and a urinary tract infection being treated with oral antibiotics. She had been diagnosed with Turner syndrome shortly after birth and had received long-term hormone replacement therapy to induce regular menstrual periods from age 18-40. She had entered menopause following the cessation of this treatment. The cessation of hormone replacement therapy had coincided with the onset of progressive urogenital symptoms. She also had congenital left renal agenesis, hypothyroidism, and diabetes. She was also taking medications for chronic anxiety disorder.

**Figure 1:**
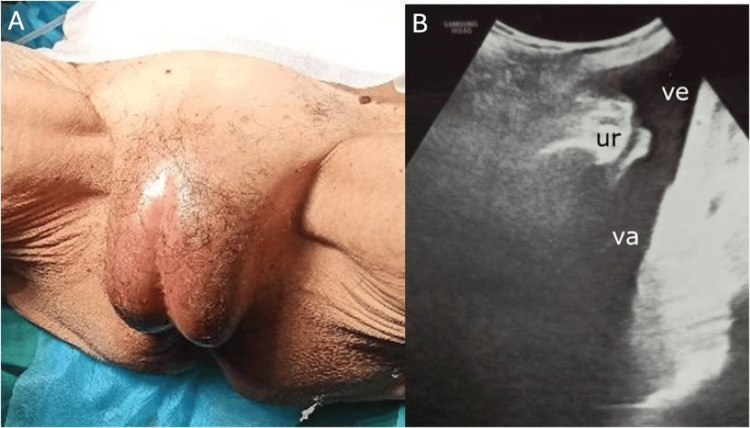
Clinical and sonographic examination A. Markedly edematous and protruding labia majora with complete midline fusion. B. Transperineal sonographic appearance of hydrocolpos and distended vulval vestibule and urethral meatus VA: vagina; VE: vestibule; UR: urethra

**Figure 2:**
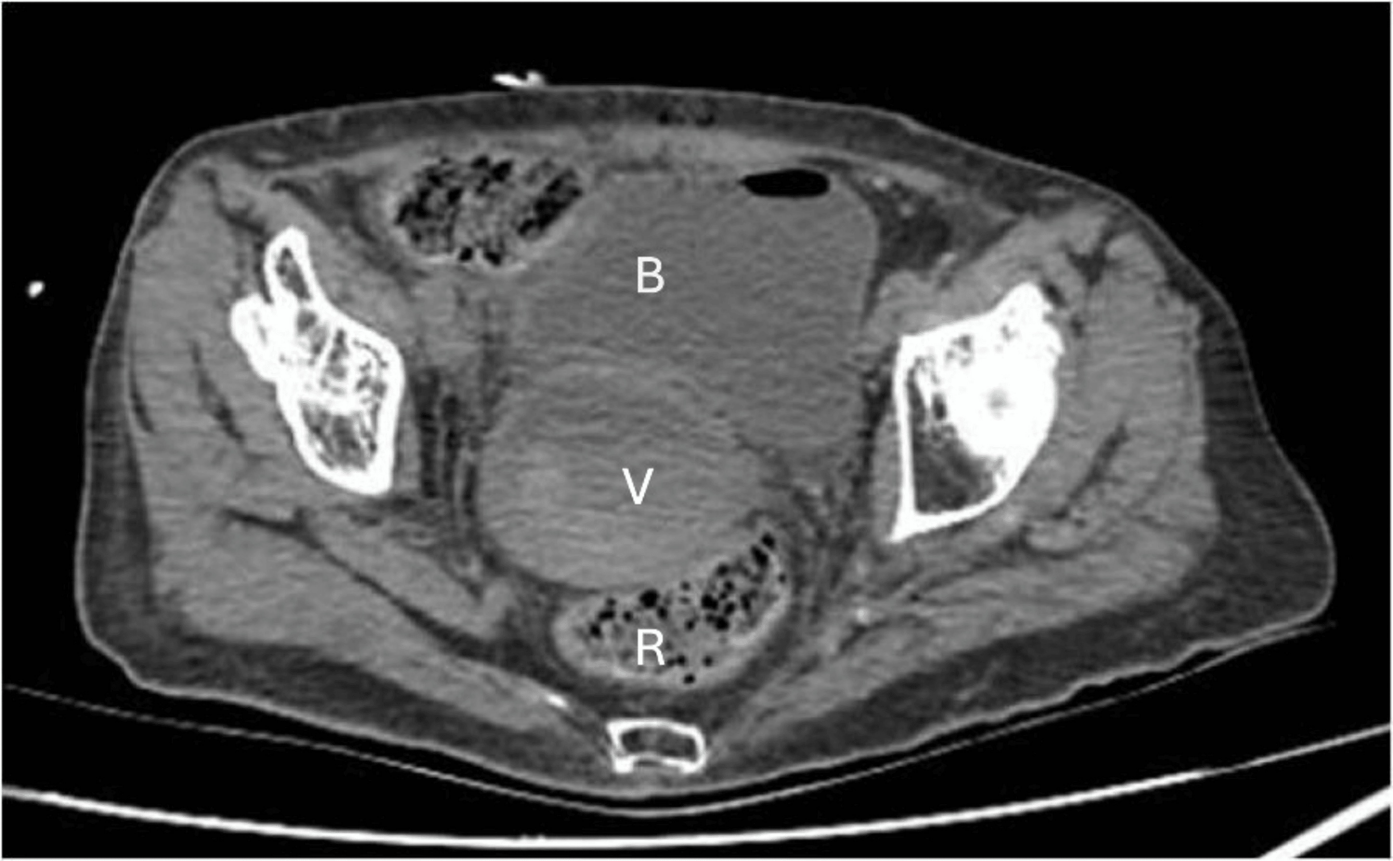
CT findings CT showing the fluid-filled vaginal cavity (V) with high-signal intensity, supporting the diagnosis of hydro-pyo-hematocolpos CT: computed tomography; B: bladder; R: rectum; V: vaginal cavity

The clinical examination of the introitus revealed markedly edematous and protruding labia majora with complete midline fusion forming a fixed raphe (Figure 1A). The clitoris, the labia minora, the urethral meatus, and the vaginal opening could not be visualized. A transperineal ultrasound revealed urine bladder distention, accumulation of echogenic fluid between the labial fusion and the vaginal opening, and hydrocolpos. The distal part of the urethra and the urethral meatus could also be visualized sonographically (Figure 1B). Visualization of the uterus was limited; however, the transabdominal approach suggested it was reduced in size with a normal appearance. CT confirmed the fluid-filled vaginal cavity with high-signal intensity, further supporting the diagnosis of hydro-pyo-hematocolpos (Figure 2).

The patient also exhibited tachycardia, tachypnea, hypotension, and a gradual impairment in mental status. The laboratory results of leukocytosis, anemia, thrombocytopenia, impaired renal function, and increased C-reactive protein were compatible with an evolving systemic inflammation and early sepsis (Table 1). The patient's severe condition and deterioration necessitated acute and advanced management.

**Table 1:** Laboratory results showing patient’s deterioration

Laboratory parameters	Patient values	Reference range
White blood cell count	29,000/μl	4,000-11,000/μl
Hemoglobin	10.3 g/dl	11.8-17.8 g/dl
Platelet count	110,000/μl	150,000-400,000/μl
Urea	424 mg/dl	15-54 mg/dl
Creatinine	3.6 mg/dl	0.9-1.6 mg/dl
C-reactive protein	20 mg/dl	Positive: >0.8 mg/dl

**Figure 3:**
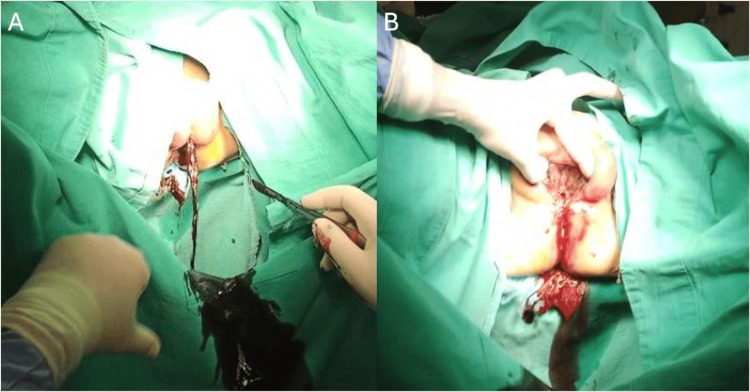
Intraoperative images A. Surgical drainage of the malodorous, purulent, and dark hemorrhagic fluid of pyo-hematocolpos; B. Restored introitus anatomy after the incision of the fibrotic labial adhesion, with visualization of the urethral meatus, the clitoris, the labia minora, and the vaginal opening

Surgical intervention was indicated, and an incision was made in the fibrotic labial adhesion under general anesthesia, leading to the drainage of a substantial volume of malodorous, purulent, and dark hemorrhagic fluid, confirming the septic nature of the retained vaginal content (Figure 3A). The whole introitus anatomy was automatically restored with visualization of the urethral meatus, the clitoris, the labia minora, and the vaginal opening (Figure 3B). A Foley catheter was placed soon after the visualization of the urethral meatus to restore urinary drainage. The patient was also administered intravenous fluids, inotropes, broad-spectrum antibiotics, and cortisol to treat her sepsis. Her condition rapidly improved, and she was discharged 14 days later, with a prescription for local estrogen cream.

## Discussion

Labial fusion or synechiae, also referred to as labial adhesion or labial agglutination, may be congenital or acquired. Acquired cases result from states of estrogen deficiency, such as prepubertal girls, geriatric postmenopausal women, or Turner syndrome, characterized by thin, membranous midline adherence of the labia forming a raphe [3-6]. Typically, the fusion originates from the posterior fourchette and advances toward the clitoris. Labial synechiae may be caused by chronic vulvovaginitis and/or chronic dampness due to urinary incontinence [4,7]. Few layers of epithelial cells may denudate from the labia minora, and apposition of the eroded areas can result in labial synechiae formation [8]. The condition's higher prevalence in children and elderly women has been attributed to a relative hypoestrogenic state [4,7,8]. Other aggravating factors include eczema, seborrhic dermatitis, lichen planus or sclerosus, recurrent urinary tract infections, and chronic inflammation due to poor hygiene and local trauma [5]. Absence of sexual activity contributes further to the development of adhesions due to reduced mechanical opening of the tissues.

Patients usually present with mild urinary or vulval symptoms, such as sexual difficulties and superficial dyspareunia, or may even be asymptomatic; however, complete labial fusion may totally conceal the vaginal introitus and cause urinary retention [4]. Our case of complete labial fusion and urine retention is a rare clinical entity, and most women have a history of incomplete voiding and dribbling of urine for several months, like our case, for which they usually seek help promptly [6]. However, in neglected cases, urinary retention and other severe complications may occur. Some cases are diagnosed late due to patients avoiding medical care or a lack of regular gynecological examinations.

As the vagina is not a sterile environment, labial fusion predisposes the woman to vaginal infections and abscesses, especially if fusion is complete. This is the mechanism behind the occurrence of pyocolpos. The accumulation of urine in the vaginal introitus and upwards into the vagina after urinary retention leads to upward infection of the genitourinary canal. Kidney infection may occur, as well as pyometra, which is a serious and potentially life-threatening condition. Even pyosalpinx as a sequela of labial fusion after menopause has been described in the literature [9]. Our patient lived alone and had a history of diabetes since early adulthood. Comorbidities, particularly diabetes, can compromise the immune system, allowing infections to worsen and unexpectedly progress to a life-threatening septic state. This likely explains why our patient’s condition deteriorated, and she was already septic upon hospital admission.

As already mentioned, during the surgical treatment of our case, a pyo-hematocolpos was observed. The blood aggregation further induced the infection, acting as a fine nutrient substrate for the vaginal bacteria. A common cause of hematocolpos or hematocolpometra is the rare congenital condition of an imperforate hymen, which leads to the accumulation of varying amounts of menstrual blood in the reproductive organs around the onset of menstruation [10]. Our patient had been in menopausal amenorrhea for 18 years. The source of the blood was unclear, as no active bleeding was observed from the introitus, vagina, or cervix following the surgical drainage of the abscess. We hypothesized that pressure or percussion trauma caused the bleeding; however, primary uterine bleeding or secondary uterine bleeding due to residual estrogen exposure could not be ruled out. This was because the endometrial thickness measured by vaginal ultrasound was borderline increased at approximately 6 mm. An endometrial curettage/biopsy was planned once the patient’s condition improved. Anyway, the MRI could not identify any pathology from the adnexa or the uterus other than a fuzziness of the endometrial contour, which could also be attributed to endometritis.

The management of mild cases of labial fusion includes topical estrogen application with or without topical steroids. If, however, no response to topical therapy is observed or the fusion is complete, causing severe complications, surgical separation under anesthesia should be performed [5]. The low recurrence rate following surgical release of labial fusion has made it an effective and safe method with negligible medium-term recurrence rates [11]. The application of topical estrogen after surgical intervention further minimizes the possibility of recurrence.

The delayed onset of symptoms in this case suggests a progressive fibrotic process, possibly exacerbated by recurrent local inflammation, chronic hypoestrogenic state, and mechanical irritation. The report highlights the importance of early recognition in these cases, as untreated adhesions may result in urinary obstruction and hemato-pyocolpos, ultimately progressing to sepsis if not promptly managed. The successful resolution of this case underscores the critical role of timely imaging, surgical intervention, hemodynamic support, and appropriate antimicrobial therapy to prevent further morbidity.

## Conclusions

Turner syndrome is characterized by gonadal dysgenesis and hypoestrogenemia, leading to marked atrophy of the genitourinary tract. This atrophy promotes the development of labial adhesions, recurrent urinary tract infections, and severe urological complications like urinary retention when the introitus becomes completely obstructed. Labial synechiae can be underdiagnosed or misdiagnosed in some cases, despite their potential impact, as affected individuals may avoid seeking medical attention. Early diagnosis, starting with a thorough medical history, and appropriate surgical treatment are critical to prevent serious complications and to improve the quality of life of these patients. Postoperative counseling must be provided to these patients to prevent fusion relapse and its unfavorable consequences.
